# Ketogenic Diet Initially Masks Symptoms of Hypercortisolism in Cushing’s Disease

**DOI:** 10.3390/metabo12111033

**Published:** 2022-10-28

**Authors:** Mary Kimberly Dugandzic, Esther-Carine Pierre-Michel, Tro Kalayjian

**Affiliations:** 1 School of Science, Marist College, Poughkeepsie, NY 12601, USA; 2 School of Arts and Sciences, Rutger’s University, New Brunswick, NJ 08901, USA; 3 Department of Medicine, Yale New Haven Health System, New Haven, CT 06510, USA

**Keywords:** low carb, carnivore, ketogenic, Cushing syndrome, Cushing disease, glucose intolerance, hypertension, obesity, metabolic health

## Abstract

Cushing’s syndrome (CS) is a diagnosis used to describe multiple causes of serum hypercortisolism. Cushing’s disease (CD), the most common endogenous subtype of CS, is characterized by hypercortisolism due to a pituitary tumor secreting adrenocorticotropic hormone (ACTH). A variety of tests are used to diagnose and differentiate between CD and CS. Hypercortisolism has been found to cause many metabolic abnormalities including hypertension, hyperlipidemia, impaired glucose tolerance, and central adiposity. Literature shows that many of the symptoms of hypercortisolism can improve with a low carb (LC) diet, which consists of consuming <30 g of total carbohydrates per day. Here, we describe the case of a patient with CD who presented with obesity, hypertension, striae and bruising, who initially improved some of his symptoms by implementing a LC diet. Ultimately, as his symptoms persisted, a diagnosis of CD was made. It is imperative that practitioners realize that diseases typically associated with poor lifestyle choices, like obesity and hypertension, can often have alternative causes. The goal of this case report is to provide insight on the efficacy of nutrition, specifically a LC diet, on reducing metabolic derangements associated with CD. Additionally, we will discuss the importance of maintaining a high index of suspicion for CD, especially in those with resistant hypertension, obesity and pre-diabetes/diabetes.

## 1. Introduction

Cushing’s syndrome (CS) is a rare disorder of hypercortisolism related to exposure to high levels of cortisol (>20 mcg/dL between 0600–0800 or >10 mcg/dL after 1600) for an extended period [1,2]. CS affects 10 to 15 people per million and is more common among those with diabetes, hypertension, and obesity [3]. The metabolic derangements associated with CS include visceral obesity, elevated blood pressure, dyslipidemia, type II diabetes mellitus (T2DM) and insulin resistance [4]. CS physical exam findings include round face, dorsal fat pad, central obesity, abdominal striae, acne, and ecchymosis [3]. Other symptoms associated with CS include low libido, headache, change in menses, depression and lethargy [2,3,5]. The most common features of CS are weight gain, which is found in 82% of cases, and hypertension, which is found in 50–85% of cases [6]. CS can be caused by exogenous glucocorticoids, known as iatrogenic CS, ectopic ACTH secretion (EAS) from sources like a small cell lung cancer or adrenal adenoma, known as EAS CS, or excess production of ACTH from a pituitary tumor, known as CD [3]. In CD, ACTH subsequently causes increased production of cortisol from the adrenal glands. CD accounts for 80–85% of endogenous cases of CS [3]. Other conditions including alcoholism, depression, severe obesity, bulimia and anorexia nervosa can lead to a Cushing-like state, although are not considered true CS [3]. Many studies have demonstrated that LC diets can ameliorate some of the most common metabolic derangements seen in CD, namely hyperglycemia, weight gain, hypertension and insulin resistance.

A LC diet is a general term for diets which lower the total carbohydrates consumed per day [4]. A ketogenic diet is a subtype of LC that is described as having even fewer carbohydrates, typically less than 30 g/day. By reducing carbohydrate intake and thus limiting insulin production, the body achieves ketosis by producing an elevated number of ketones including β-hydroxybutyric acid, acetoacetic acid, and acetone, in the blood [7]. A carnivore diet, a specific type of a ketogenic diet, is defined as mainly eating animal food such as meat, poultry, eggs and fish. Contrarily, a standard American diet (SAD) is defined as a diet high in processed foods, carbs, added sugars, refined fats, and highly processed dairy products [8]. There are several therapeutic applications for LC diets that are currently supported by strong evidence. These include weight loss, cardiovascular disease, T2DM, and epilepsy. LC diets have clinical utility for acne, cancer, polycystic ovary syndrome (PCOS), and neurologic deficits [9].

In this case report, the patient endorsed initially starting a LC diet to address weight gain and high blood sugars that he noted on a glucometer. The patient noted a 35 pounds (lbs.) weight loss over the first 1.5 years on his LC diet, as well as improved blood pressure and in his overall health. He then adopted a carnivore diet but found that weight loss was difficult to maintain, although his body composition continued to improveand his clothes fit better. Later, he noted that his blood pressure would at times be poorly controlled despite multiple medications and strict dietary adherence. The patient reported “being in despair” and “not trusting his doctors” because they did not understand how much his diet had helped him. Despite strict adherence, his symptoms of insulin resistance and hypertension persisted. In this report, we will describe how his symptoms of CD were ameliorated by the ketogenic diet. This case report also highlights that when patients are unable to overcome hormonal pathology, clinicians should not blame patients for lack of adherence to a diet, but instead understand the need to evaluate for complex pathology.

## 2. Detailed Case Description

A male patient in his thirties, of Asian descent, had a past medical history of easy bruising, central obesity, headaches, hematuria, and hypertension and past family medical history of hypertension in his father and brother. In 2015, he was at his heaviest weight of 179 lbs. with a body mass index (BMI) of 28 kg/m^2^, placing him in the overweight category (25.0–29.9 kg/m^2^). At that time the patient reported he was following a SAD diet and was active throughout the day. The patient stated he ate a diet of vegetables, fruits and carbohydrates, but he was not able to lose weight. The patient stated that he switched to a LC diet, to address weight gain and hyperglycemia, and he reported that he lost approximately 35 lbs. in 1.5 years. The patient described his LC diet as eating green leafy vegetables, low carb fruits, fish, poultry, beef and dairy products. The patient then later switched to a carnivore diet. He noted despite aggressively adhering to his diet, that his weight-loss had plateaued, although his waist circumference continued to decrease. The patient noted his carnivore diet consisted of eating a variety of different meats, poultry, fish and eggs.

The metabolic markers seen in Table 1 were obtained after the patient had started a carnivore diet. The patient’s blood glucose levels decreased overtime despite impaired glucose metabolism being a known side effect of hypercortisolism [4]. The patient’s high-density lipoprotein (HDL) remained in a healthy range (40–59 mg/dL) and his triglycerides stayed in an optimal range (<100 mg/dL), despite dyslipidemia being a complication of CD [4]. When the patient was consuming a SAD diet, he was not under the care of a physician and was unable to provide us with previous biomarkers.

Despite strict adherence to his diet and initial improvement in his weight, his blood pressure and his blood sugar levels, in October of 2021 the patient was admitted to the hospital for hypertensive urgency, with a blood pressure of 216/155. His complaints at the time were unexplained ecchymosis, hematuria and significant headaches that were resistant to Excedrin (acetaminophen-aspirin-caffeine) use. At the hospital, the patient underwent a computed tomography (CT) scan of the head and radiograph of the chest, and both images were negative for acute pathology. During his hospital admission, the patient denied any changes in vision, chest pain or edema of the legs. Ultimately, the patient was told to eat a low-salt diet and to follow-up with a cardiologist. At discharge, the patient was placed on hydrochlorothiazide, labetalol, amlodipine and lisinopril. The patient was then seen by his primary care physician in November of 2021 and his urinalysis at that time showed 30 mg/mL (Negative/Trace) of protein in his urine, without hematuria. The patient’s primary care physician discontinued his hydrochlorothiazide and started the patient on furosemide. Additionally, the primary care physician reinforced cutting out salt and limiting his calories to prevent any further weight gain, which his physician explained would contribute further to his hypertension. He was referred to hematology and oncology in November of 2021 for his symptoms of hematuria and abnormal ecchymosis to his abdomen, thighs and arms. The patient’s coagulation and platelet counts were normal, and his symptoms were noted to be improving. His hematuria and ecchymosis were attributed to his significant Excedrin use from the past 1–2 months, secondary to his headaches, and their anti-platelet effect. It was noted that the patient had significant hemolysis during his hospital admission. However, in his follow up examination, there were no signs of hemolysis, and it was attributed to his hypertensive urgency. Again, a low-salt, calorie-limited diet was recommended. The patient was referred to cardiology where he was evaluated for secondary hypertension, because despite his weight loss and his strict adherence to his diet, his blood pressure was still uncontrolled on multiple medications. He had a normal echocardiogram and renal ultrasound which showed no signs of renal artery stenosis bilaterally. At that time the patient’s serum renin, aldosterone and urine metanephrine levels were all normal. His cardiologist increased his lisinopril, and continued him on amlodipine, furosemide and labetalol and reinforced the recommendations of lowering his salt and preventing weight gain.

**Figure 1:**
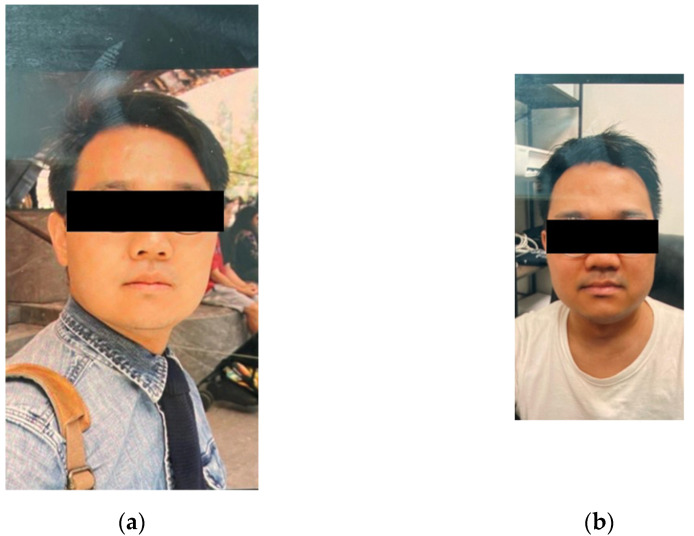
The patient’s progression of moon facies, (**a**) photo from 2019 after initial weight loss (**b**) photo from office visit in 2022.

**Figure 2:**
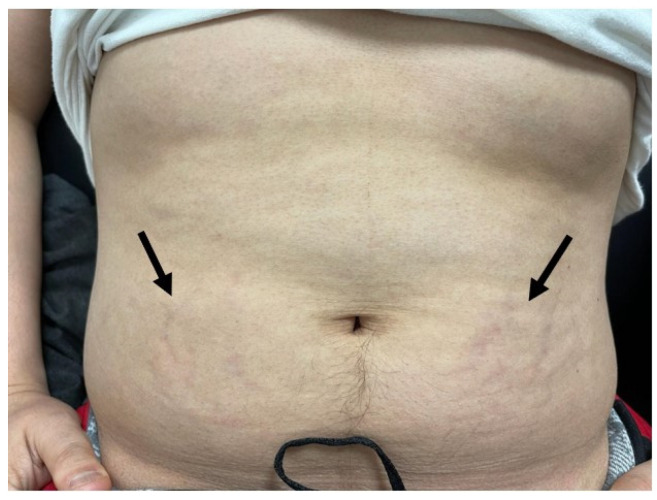
The arrows demonstrate early striae visualized on the lower abdomen bilaterally, unclear in image due to poor office lighting.

The patient first contacted our office in January of 2022. At that time his blood pressure was noted to be 160/120 despite being compliant with current blood pressure medications. The patient reported strict adherence to his carnivore diet by sharing his well-documented meals on his social media accounts. Given the persistent symptoms, despite his significant change in diet and weight loss, we were concerned that a hormonal etiology may be driving his symptoms. The patient was seen in-person, in our office, in March of 2022. At the request of the patient, we again reviewed his social media profile to assess his meal choices and diet. While the patient was eager to show us his carnivore meals, what we incidentally noted in his photos was despite weight loss and strict diet adherence, he had developed moon facies (Figure 1a,b). On the physical exam, we noted his prominent abdominal striae (Figure 2). Several screening tests for Cushing’s syndrome were ordered. A midnight salivary cortisol was ordered, with values of 0.884 ug/dL (<0.122 ug/dL) and 0.986 ug/dL (<0.122 ug/dL) and a urinary free cortisol excretion (UFC) was ordered, with values of 8.8 ug/L (5–64 ug/L). At this point our suspicion was confirmed that the patient had inappropriately elevated cortisol.

**Figure 3:**
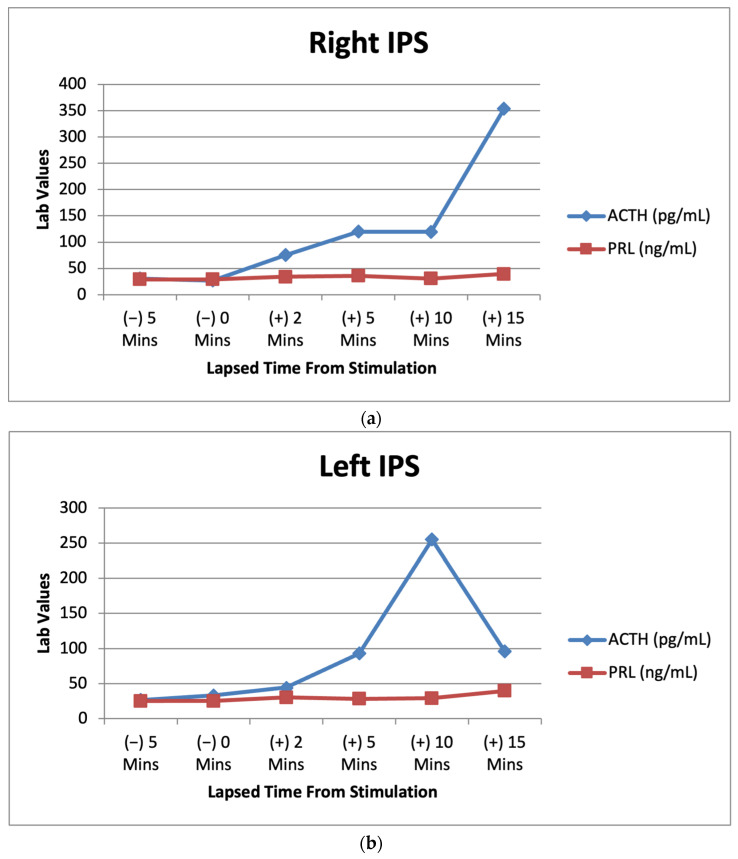

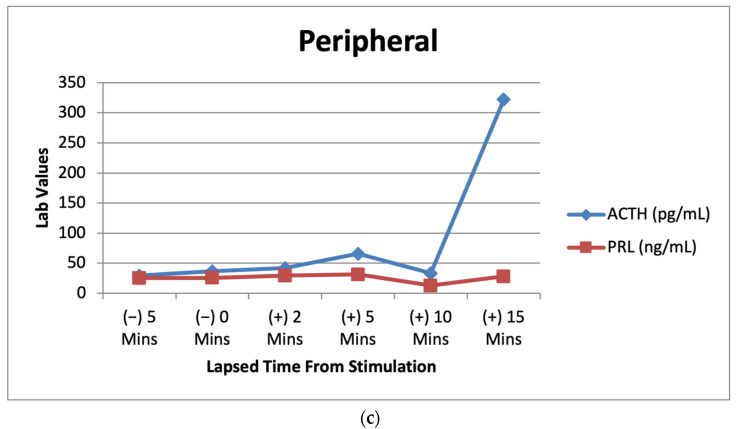
(**a**) Right IPS venous sampling values for ACTH and prolactin after CRH stimulation over multiple time intervals. (**b**) Left IPS venous sampling values for ACTH and prolactin after CRH stimulation over multiple time intervals. (**c**) Peripheral sampling values for ACTH and prolactin after CRH stimulation over multiple time intervals.

Based on screening tests and significant physical exam findings, we referred the patient to endocrinology for a low dose dexamethasone suppression test (DST). They performed a low dose DST revealing a dehydroepiandrosterone (DHEA) of 678 ug/dL (89–427 ug/dL) and ACTH of 23.9 pg/mL (7.2–63.3 pg/mL). The low dose DST and midnight salivary cortisol were both positive indicating hypercortisolism. To begin determining the source of hypercortisolism, the plasma ACTH was evaluated and was 27.2 pg/mL (7.2–63.3 pg/mL). While ACTH was within normal range, a plasma ACTH > 20 pg/mL is suggestive of ACTH-dependent CS, so a magnetic resonance imaging (MRI) of the brain was ordered [2]. The MRI revealed a 4 mm heterogeneous lesion in the central pituitary gland which is suspicious of a cystic microadenoma. To confirm that a pituitary tumor was the cause of the patient’s increased cortisol, the patient was sent for inferior petrosal sinus sampling (IPSS). The results of the IPSS indicated an increase in ACTH in both inferior petrosal sinuses and peripheral after corticotropin-releasing hormone (CRH) stimulation (Figure 3a–c), which was consistent with hypercortisolism.

Lab results from the patient’s IPSS venous sampling can be seen above. The graphs depict the lab values of ACTH (7.2–63.3 pg/mL) and prolactin (PRL) (2.1–17.7 ng/mL) before and after CRH stimulation during IPSS. PRL acts as a baseline to indicate successful catheterization in the procedure [10].

Using the ACTH levels from our patient’s IPSS we calculated a ratio of inferior petrosal sinus to peripheral (IPS:P). These results can be seen below (Table 2). The right IPS:P was calculated as 3.60 at 10 min and the left IPS:P as 7.65 at 10 min. These ratios confirmed that the hypercortisolism was due to the pituitary tumor, as it is higher than the 3:1 ratio necessary for diagnosis of CD [11]. The patient is currently scheduled to undergo surgical resection of the pituitary microadenoma.

## 3. Clinical Evaluation for CS

In this case, the patient presented with uncontrolled hypertension, weight gain despite a strict diet, hyperglycemia, abdominal striae and moon facies. Despite evaluation, both inpatient and outpatient, a diagnosis of CS was not yet explored. When CS is suspected based on clinical findings, the use of exogenous steroids must first be excluded as it is the most common cause of hypercortisolism [3]. If there is still concern for CS, there are three screening tests that can be done which are sensitive but not specific for hypercortisolism. The screening tests include: a 24-h UFC, 2 late night salivary cortisol tests, low dose (1 g) DST [3]. To establish the preliminary diagnosis of hypercortisolism two screening tests must be abnormal [2].

The first step to determine the cause of hypercortisolism is to measure the plasma level of ACTH. Low values of ACTH < 5 pg/mL indicate the cause is likely ACTH-independent CS and imaging of the adrenal glands is warranted as there is a high suspicion of an adrenal adenoma [2,3]. When the serum ACTH is elevated >/20 pg/mL it is likely an ACTH-dependent form of CS [2]. To further evaluate an ACTH-dependent hypercortisolism, an MRI should be obtained as there is high suspicion that the elevated cortisol is coming from a pituitary adenoma. If there is a pituitary mass >6 mm there is a strong indication for the diagnosis of CD [2]. However, pituitary tumors can be quite small and can be missed on MRIs in 20–58% of patients with CD [2]. If there is still a high suspicion of CD with an inconclusive MRI, a high dose DST (8 g) is done. Patients with CD should not respond and their ACTH and DHEA, a steroid precursor, should remain high. Similarly, CRH stimulation test is done and patients with CD should have an increase in ACTH and/or cortisol within 45 min of CRH being given. If the patient has a positive high-dose DST, CRH-stimulation test and an MRI with a pituitary tumor >6 mm no further testing is needed as it is likely the patient has CD [2]. If either of those tests are abnormal, the MRI shows a pituitary tumor < 6 mm, or there is diagnostic ambiguity, the patient should undergo IPSS with ACTH measurements before and after the administration of CRH [4]. IPSS is the gold standard for determining the source of ACTH secretion and confirming CD. In this invasive procedure, ACTH, prolactin, and cortisol levels are sampled prior to CRH stimulation and after CRH stimulation. PRL acts as a baseline to indicate successful catheterization in the procedure [12]. To confirm CD, a ratio of IPS:P is calculated for values prior to and after CRH stimulation. A peak ratio greater than 2.0 before CRH stimulation or a peak ratio greater than 3.0 after CRH stimulation is indicative of CD. In comparing the right and left petrosal sinus sample, an IPS:P ratio greater than 1.4 suggests adenoma lateralization. However, due to high variability, IPSS should not be used for diagnosing lateralization [13].

## 4. Discussion

Surgical intervention remains the primary treatment for CD [4]. However, remission is not guaranteed as symptoms and metabolic diseases have been shown to persist afterwards. In the literature it has been shown that nutrition can have a powerful impact on suppressing, or even reversing metabolic disorders and comorbidities associated with CD. A LC diet has been shown to promote significant weight loss, reduce hypertension, improve dyslipidemia, reverse T2DM and improve cortisol levels (2, 14–15, 18–21).

There are reports of weight loss on a LC diet in the literature. A LC significantly reduced weight and BMI of 30 male subjects [14]. In a group of 120 participants over 24 weeks who followed a LC versus low fat (LF) diet, showed a greater weight loss in the LC group vs. the LF group [15]. Patients diagnosed and treated for CD found that their weight remained largely unchanged even after treatment [6]. In many cases, surgical treatment does not always resolve the associated comorbidity of central adiposity in CD. In such cases, a LC diet can be used before, during and after treatment, as an adjunct, to decrease associated weight gain and comorbidities.

Nutritional intervention can be a powerful adjunct to reduce comorbidities associated with CD. As seen in this case report, the patient’s symptoms of CD, especially hypertension and weight gain, improved with dietary changes despite him having a pituitary microadenoma. Multiple studies showed that a LC diet was able to decrease blood pressure parameters. In a group of 120 participants over 24 weeks who followed a LC versus a LF diet showed a greater decrease in both systolic and diastolic blood pressure in the LC group vs. the LF group [15]. Other literature which studied the effect of a LC diet on hypertension demonstrated the reduction of blood pressure and is thought to be due to ketogenesis. It is thought the production of ketones have a natriuretic effect on the body therefore lowering systemic blood pressure [16].

A LC diet improves lipid profiles and inflammatory markers associated with metabolic syndrome [14]. Literature shows that a LC diet has a greater impact on decreasing triglyceride levels and increasing HDL levels, when compared to a LF diet [15]. Triglyceride levels in patients in CD remission remained high [17]. Therefore, it can be hypothesized that a LC diet would be beneficial, in addition to standard CD treatment, to lower the associated comorbidity of hypertriglyceridemia and metabolic syndrome.

Insulin resistance, a precursor to T2DM, is a common comorbidity of hypercortisolism which can be treated with a LC diet. One study showed that in subjects with T2DM, a decrease in A1c and a reduction in antidiabetic therapy were seen with consumption of a LC diet [18]. Additionally, a cohort of 9 participants following a LC diet were able to collectively lower their A1c on average by 1% while concurrently discontinuing various antidiabetic therapies including insulin [19].

Literature shows that a LC diet can minimize systemic cortisol levels through various mechanisms. Current treatment of CD includes medications which block cortisol production and/or cortisol secretion [2]. LC can imitate similar results seen through medication intervention for CD. Carbohydrate restriction can lower cortisol levels, as carbohydrates stimulate adrenal cortisol secretion and extra-adrenal cortisol regeneration [4]. A ketogenic diet can lower the level of ghrelin, a peptide produced in the stomach that has orexigenic properties [20,21]. Literature shows that ghrelin increases levels of serum cortisol [22]. Therefore, implementing a ketogenic diet would decrease ghrelin, and subsequently minimize the effects of increased ghrelin on serum cortisol. A LC diet decreases visceral fat which itself is an endocrine organ and can increase the synthesis of cortisol [14]. Therefore, decreasing visceral fat also decreases the production of cortisol. A LC was shown to significantly reduced weight, BMI and cortisol levels of 30 obese male subjects [14]. Further, a LC diet excludes foods with a high glycemic index which cause increased stress on the body which subsequently leads to the activation of the hypothalamic-pituitary-axis which causes increased levels of cortisol [14].

This case report illustrated how a LC diet was initially successful at ameliorating the patient’s associated symptoms of hypertension and obesity, making his diagnosis of CD go undetected. Literature shows that while the prevalence of CS on average is a fraction of a percent, it is much higher among patients with poorly controlled diabetes, hypertension and early onset osteoporosis [3]. Two hundred patients with diabetes mellitus were studied and 5.5% were found to have CS [23]. Another study discovered that in subjects with CD, 36.4% were found to have hyperlipidemia, 73.1% with hypertension, and 70.2% with impaired glucose metabolism [17]. It can be concluded that a higher index of suspicion and lower threshold for screening for CS may be necessary in obese and diabetic patient populations. A lower threshold for screening can allow for earlier diagnosis for many patients, and therefore provide better outcomes for those diagnosed with CS.

It is important for clinicians to consider alternative pathology for patients combating metabolic derangements. As depicted in this case, the patient lost 35 lbs. while on a LC diet, despite having hypercortisolism, presumably for months to years prior to the diagnosis of his condition. The patient noted a tendency to gain weight, have elevated blood sugar and blood pressure which prompted him to begin self-treatment with increasingly strict carbohydrate restriction. The patient was able to keep his symptoms of hypercortisolism managed, potentially making the diagnosis difficult for his team of clinicians. From a diagnostic perspective, it’s important to understand that strict dietary adherence can have profound impacts on even the most severe hormonal pathology. Ultimately, this case serves as a reminder of the power of nutrition to address metabolic derangements and simultaneously as a reminder to diagnosticians to never rely on lack of dietary adherence as a reason for persistent metabolic symptoms. The reflexive advice to “not gain weight” and “lower salt intake” in retrospect appears both dogmatic and careless. In this case, the patient had seen several doctors and was even hospitalized and yet his disease state remained unclear and the dietary messaging cursory.

## 5. Conclusions

Many chronic diseases, including diabetes, hypertension and obesity, are generally thought to be caused by dietary and lifestyle choices. However, as exemplified in this report underlying medical problems, such as endocrine disorders, can be the cause of such metabolic derangements. It is critical that practitioners consider other causes of metabolic derangements, as assuming that they are due to poor dietary adherence, can allow them to go undiagnosed. While there is extensive literature on LC diets and their effect on the metabolic derangements associated with hypercortisolism, there needs to be further research on LC as an adjunctive therapy to conventional CD treatment. Ultimately, nutrition can have a powerful impact on suppressing, or even reversing metabolic disorders. As depicted in this case study, a LC diet is powerful enough to temporarily suppress symptoms of CD.

## Figures and Tables

**Table 1:** Patient’s metabolic markers on a carnivore diet. Glucose (70 to 99 mg/dL), total cholesterol (desirable <200 mg/dL, borderline high 200–239 mg/dL, high >239 mg/dL), triglycerides (optimal: <100 mg/dL), HDL (low male: <40 mg/dL), low density lipoprotein (LDL) (Optimal: <100 mg/dL).

	8 Febuary 2022	4 November 2021	30 October 2021
Glucose (mg/dL)	99	105	115
Total cholesterol (mg/dL)	275	275	284
Triglyceride (mg/dL)	88	83	63
HDLc (mg/dL)	50	56	71
LDLc (mg/dL)	210	210	206

**Table 2:** Right and left petrosal sinus to peripheral serum ACTH ratios.

	Right Ratio	Left Ratio
(−) 5 min	1.05	0.90
0 min	0.73	0.90
(+) 2 min	1.81	1.06
(+) 5 min	1.82	1.41
(+) 10 min	3.60	7.66
(+) 15 min	1.10	0.30

## Data Availability

The data presented in this study are available in article.
